# Spatio-temporal analysis of female breast cancer incidence in Shenzhen, 2007–2012

**DOI:** 10.1186/s40880-015-0013-y

**Published:** 2015-05-14

**Authors:** Hai-Bin Zhou, Sheng-Yuan Liu, Lin Lei, Zhong-Wei Chen, Ji Peng, Ying-Zhou Yang, Xiao-Li Liu

**Affiliations:** Shenzhen Center for Chronic Disease Control, Shenzhen, Guangdong 518020 P. R. China; Shenzhen Nanshan Center for Chronic Disease Control, Shenzhen, Guangdong 518054 P. R. China

**Keywords:** Breast cancer, Spatial analysis, Spatial autocorrelation, Spatio-temporal clustering

## Abstract

**Introduction:**

Breast cancer is a leading tumor with a high mortality in women. This study examined the spatio-temporal distribution of the incidence of female breast cancer in Shenzhen between 2007 and 2012.

**Methods:**

The data on breast cancer incidence were obtained from the Shenzhen Cancer Registry System. To describe the temporal trend, the average annual percentage change (AAPC) was analyzed using a joinpoint regression model. Spatial autocorrelation and a retrospective spatio-temporal scan approach were used to detect the spatio-temporal cluster distribution of breast cancer cases.

**Results:**

Breast cancer ranked first among different types of cancer in women in Shenzhen between 2007 and 2012 with a crude incidence of 20.0/100,000 population. The age-standardized rate according to the world standard population was 21.1/100,000 in 2012, with an AAPC of 11.3%. The spatial autocorrelation analysis showed a spatial correlation characterized by the presence of a hotspot in south-central Shenzhen, which included the eastern part of Luohu District (Donghu and Liantang Streets) and Yantian District (Shatoujiao, Haishan, and Yantian Streets). Five spatio-temporal cluster areas were detected between 2010 and 2012, one of which was a Class 1 cluster located in southwestern Shenzhen in 2010, which included Yuehai, Nantou, Shahe, Shekou, and Nanshan Streets in Nanshan District with an incidence of 54.1/100,000 and a relative risk of 2.41; the other four were Class 2 clusters located in Yantian, Luohu, Futian, and Longhua Districts with a relative risk ranging from 1.70 to 3.25.

**Conclusions:**

This study revealed the spatio-temporal cluster pattern for the incidence of female breast cancer in Shenzhen, which will be useful for a better allocation of health resources in Shenzhen.

## Background

Breast cancer is a leading tumor among women worldwide, with an incidence that has displayed a gradual increasing trend in many countries over the past 30 years [1]. According to the GLOBOCAN 2012 released by the International Agency of Research on Cancer (IARC), there were approximately 1.7 million newly diagnosed cases of breast cancer and 0.5 million deaths in women worldwide in 2012 [2]. Moreover, the age-standardized rate (ASR) of mortality in developed countries was 1.8 times that in developing countries [2].

In China, breast cancer ranked as the most common type of cancer and the fifth leading cause of cancer deaths among women; the ASR of incidence was estimated to be 23.2/100,000, and the ASR of mortality was approximately 4.9/100,000. Although the incidence of breast cancer among Chinese women was relatively lower than that in developed countries, an increasing trend has been witnessed in recent years [3].

At present, it is widely believed that the development of breast cancer can be attributed to genetic factors, lifestyle changes, and environmental exposure, among which environmental factors and individual behaviors are believed to be factors that can be modified to prevent breast cancer [4]. However, the risk factors for breast cancer might be different between the Chinese and Western populations [5]. Therefore, studies are warranted to explore the causes of breast cancer in China.

Certain personal characteristics, such as genetic inheritance and lifestyle, have been explored in a few previous studies [6,7], but spatial distribution information is rare. Such analysis will be useful in exploring the risk factors associated with the distribution patterns of breast cancer, which can provide not only etiologic clues but also decision-making information for the effective implementation of breast cancer prevention and health promotion.

This study aimed to explore the spatio-temporal distribution pattern of female breast cancer using the cancer information obtained from the Shenzhen Cancer Registry System.

## Methods

### Data source

The data on the breast cancer cases in this study were obtained from the Shenzhen Cancer Registry System, which was established in 1998 and covers all permanent residents in Shenzhen city. In this system, all of the breast cancer cases diagnosed in qualified hospitals (defined as the hospitals with tumor diagnosis and treatment qualifications) were requested to be reported with a unified tumor reporting card according to the International Classification of Diseases, 10th revision (ICD-10). In addition, these data were supplemented by the Shenzhen Death Registration System to account for potentially under-reported cases.

The incidence of breast cancer was estimated according to the population data from the statistical yearbook of Shenzhen with age groups calculated using the 2010 census information from Shenzhen City [8]. The data from the Shenzhen Cancer Registry System between 2007 and 2012 were included in this study. It should be noted that there was a lag of 1 year for the cancer registries to verify and clean the registered data before the data were available for analysis. For each case, information on the place of residence was classified according to the minor civil division with the ratio of 1:10,000 based on the geographic information in the Shenzhen administrative division map provided by the National Geographic Center of China.

### Quality control

The percentage of cases with morphologic verification (MV%), percentage of cancer cases identified with death certificates only (DCO%), and percentage of other and unspecified cases (O&U%) were used to evaluate the completeness, validity, and reliability of the cancer registration data. According to the acceptable criteria of the IARC, the following standards should be reached: an MV% higher than 66%, a DCO% lower than 15%, and an O&U% lower than 5%.

The overall values of MV%, DCO%, and O&U% were 90.04%, 1.25%, and 2.84%, respectively. The quality evaluation for each cancer registration is presented in Table 1.

**Table 1:** **Quality evaluation of breast cancer registration at each district of Shenzhen between 2007 and 2012**

**District**	**MV%**	**DCO%**	**O&U%**
Luohu	93.45%	0.89%	1.85%
Nanshan	92.57%	0.76%	1.43%
Futian	93.75%	0.72%	1.56%
Yantian	91.57%	1.08%	2.07%
Bao’an	89.72%	1.57%	2.93%
Longgang	87.48%	1.82%	2.89%
Guangming	89.71%	1.37%	2.68%
Pingshan	88.45%	1.24%	2.71%
Longhua	87.51%	1.37%	2.84%
Dapeng	86.45%	1.45%	3.04%
Total	90.04%	1.25%	2.84%

MV%, percentage of cases with morphologic verification; DCO%, percentage of cancer cases identified with death certificates only; O&U%, percentage of other and unspecified cases.

### Spatial clusters

A spatial cluster analysis of breast cancer cases was performed using spatial autocorrelation [9]. A spatial cluster model of the overall area was estimated via the global spatial autocorrelation index Moran’s *I* (global indicators of spatial association, GISA); the cluster type and exact position were examined using local Moran’s *I* (local indicators of spatial association, LISA). The values of global Moran’s *I* ranged from −1 to 1, and the greater the absolute correlation value, the stronger the spatial autocorrelation. When *I* > 0, the disease distribution is positive for spatial autocorrelation and vice versa. A high *I* value (hotspot, high-high) or low *I* value (coldspot, low-low) exists when the LISA statistics are positive, and different observations (low-high) are present when the LISA statistics are negative.

### Spatio-temporal scan

The spatio-temporal cluster detection test for breast cancer incidence was retrospectively performed using spatial scan statistics. The scan parameters were as follows: the time range was between 2007 and 2012; the time interval was 1 year; the potential population risk was 10%; and the number of Monte Carlo simulations was restricted to 999 times [10]. Then, the log likelihood ratio (*LLR*) was obtained from the actual incidence and theoretical incidence computed by the Poisson distribution in each scan window. The formula was as follows: *LLR* = log(*c*/*n*)^c^ [(*C* - *c*) / (*C* - *n*)]^(*C*-*c*)^ (where *C* is the total number of cases, *c* is the number of cases in the scanning window, and *n* is the expected number of cases in the active scanning window). The scanned area involving the maximum *LLR* value with statistical significance was defined as a Class 1 cluster, and the other scanned areas containing only *LLR* values with statistical significance were identified as Class 2 clusters. The relative risk (*RR*) was calculated as the ratio of the incidence inside a cluster area to the incidence outside a cluster area [10].

### Statistical analysis

The ASR according to the Chinese population (CASR) was estimated using the national 1982 census information, and the ASR according to the world standard population (WASR) was estimated using Segi’s world standard population. The descriptive analysis was carried out using Stata Version 12.0 (Stata Corp., College Station, TX, USA). The temporal trend of incidence was evaluated via the annual percentage change (APC) and the average annual percentage change (AAPC) using the joinpoint regression model [11]. The spatial cluster analysis was performed by the hypothesis testing of *z* statistics for space aggregation indices using Geoda 1.6 software (GeoDa Center, Tempe, AZ, USA) [12]. The spatio-temporal scan analysis was achieved with the SaTScan 9.3 program developed by National Cancer Institute (NCI, Boston, MA, USA) [10].

## Results

### Basic information

Between 2007 and 2012, a total of 5,511 breast cancer cases were reported in Shenzhen, which accounted for approximately 16.79% of the cancer cases among women, and breast cancer ranked as the most common type of female cancer. The total crude incidence of breast cancer was 20.0/100,000 with a CASR of 29.1/100,000 and a WASR of 21.1/100,000.

**Figure 1:**
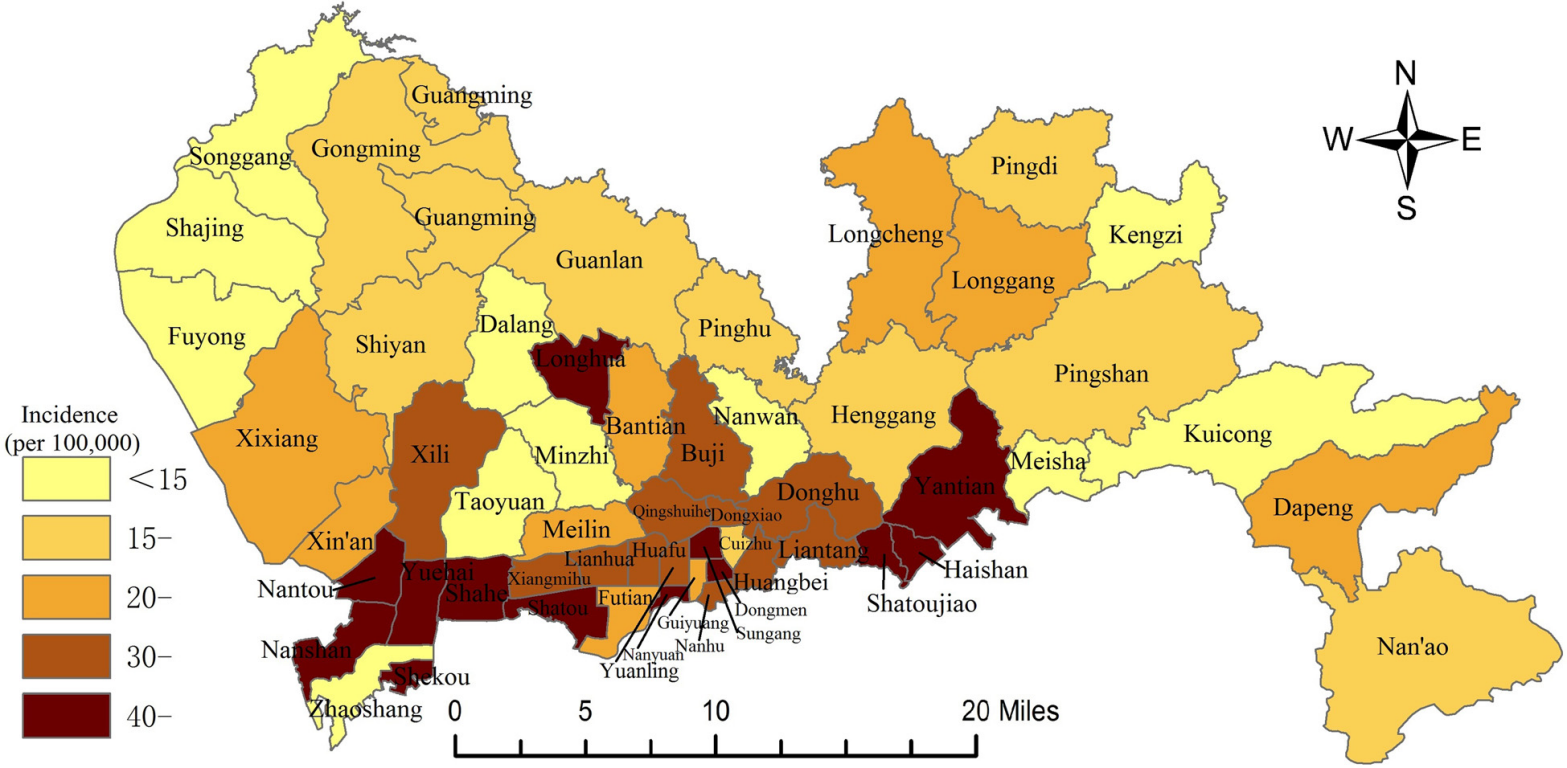
Geographic distribution of female breast cancer incidence in Shenzhen, 2007–2012.

The geographic distribution of the incidence of breast cancer was characterized by a higher incidence in urban areas than in rural areas, with the lowest incidence on Dalang Street (WASR: 4.1/100,000) in Longhua District and the highest incidence on Shatoujiao Street (WASR: 90.5/100,000) in Yantian District. The basic information is shown in Figure 1.

### Temporal trend

**Figure 2:**
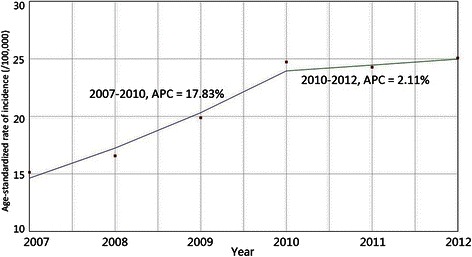
Joinpoint analysis of the age-standardized rate of female breast cancer incidence in Shenzhen, 2007–2012. APC, annual percentage change.

The quantitative analysis of the temporal trend of breast cancer incidence via the joinpoint regression model suggested that the ASR of breast cancer incidence had an increasing trend with an AAPC of 11.3%. Additionally, the temporal trend could be divided into two periods: a rapid growth period (2007–2010) with an APC of 17.83% followed by a stable growth period (2010–2012) with an APC of 2.11% (*F* = 3.849, *P* = 0.02) (Figure 2).

### Global spatial autocorrelation

The global spatial autocorrelation analysis of the cumulative ASR of incidence in Shenzhen between 2007 and 2012 showed an Moran’s *I* index of 0.372 (*z* = 5.592, *P* < 0.01). According to the results from the temporal trend analysis, the spatial distribution of breast cancer incidence can be divided into a rapid growth period (2007–2010) and a stable growth period (2010–2012). The global autocorrelation analysis also showed that Moran’s *I* index was still robust at each stage (Moran’s *I* index = 0.391, *z* = 4.891, *P <* 0.01 for the rapid growth period; Moran’s *I* index = 0.305, *z* = 4.107, *P* < 0.01 for the stable growth period), indicating that the occurrence of breast cancers exhibits spatial clustering by clusters of streets with high and low incidences.

### Local spatial autocorrelation

**Figure 3:**
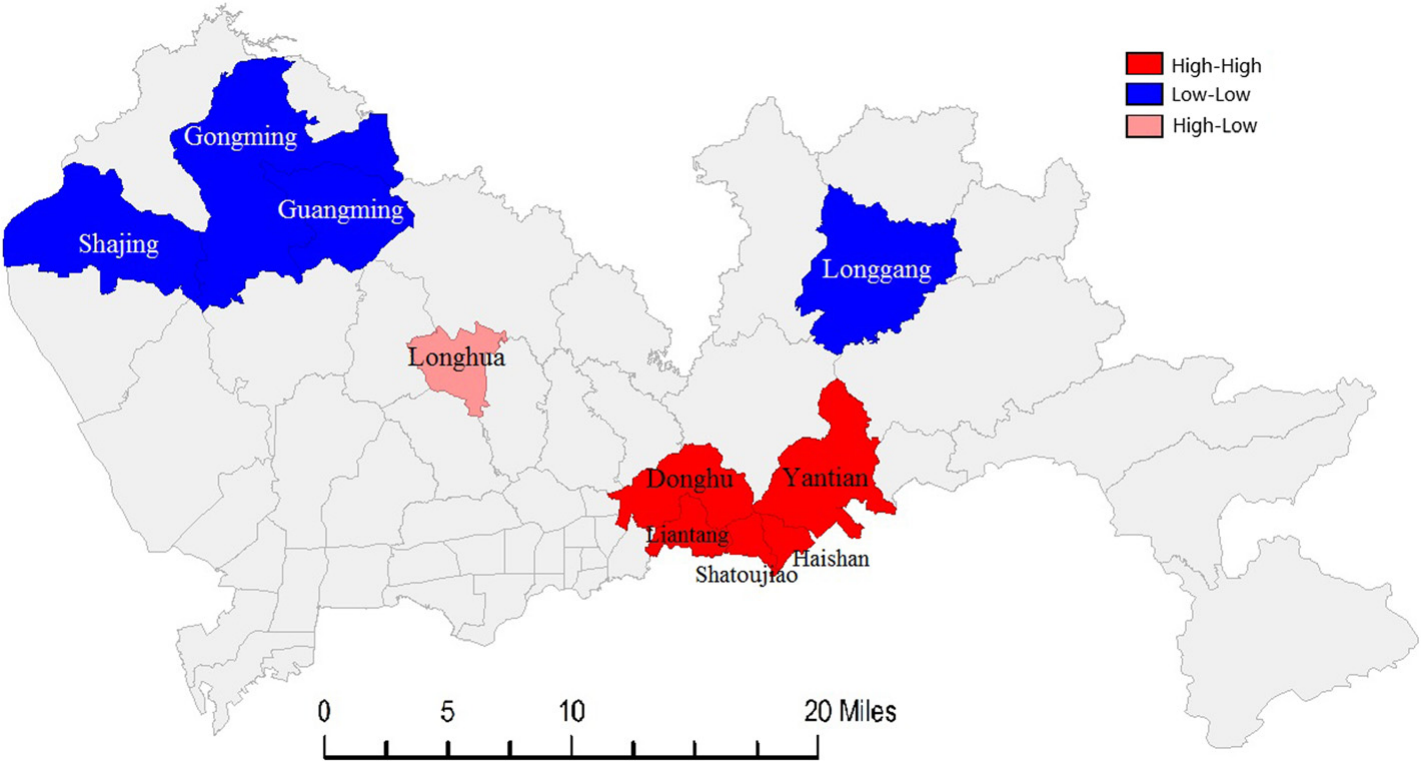
Local hotspot map for female breast cancer incidence in Shenzhen, 2007–2012.

The local autocorrelation analysis of the cumulative ASR of incidence in Shenzhen between 2007 and 2012 showed the presence of local hotspots or coldspots with a local Moran’s *I* index of 0.372 (*z* = 5.185, *P* < 0.01). Moreover, the LISA visualization analysis demonstrated the presence of a hotspot (high-high) of breast cancer incidence in south-central Shenzhen, which included eastern Luohu District (Donghu and Liantang Streets) and Yantian District (Shatoujiao, Haishang, and Yantian Streets), and a coldspot (low-low) that included Longgang, Gongming, and Guangming Streets (Figure 3).

### Spatio-temporal cluster

**Figure 4:**
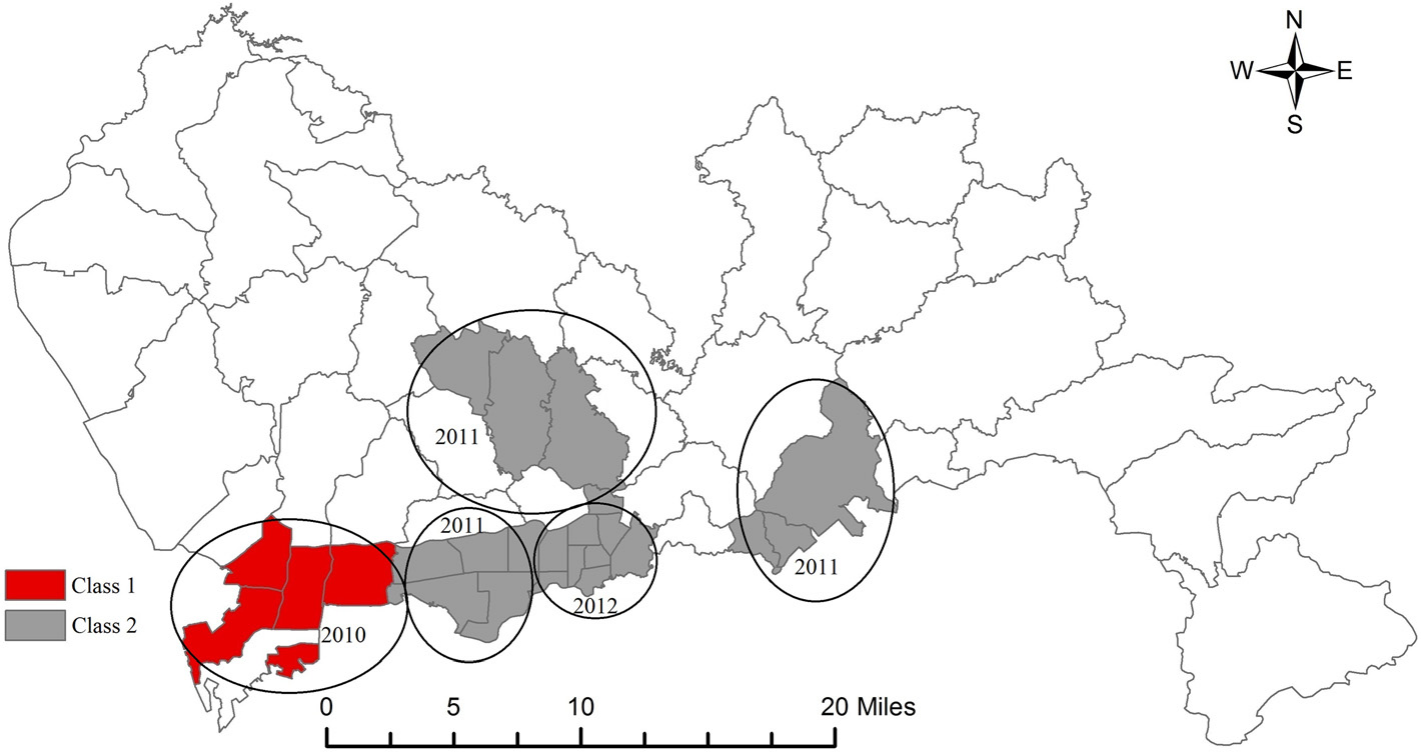
Spatio-temporal cluster map of female breast cancer incidence in Shenzhen, 2007–2012.

The data of breast cancer incidence in Shenzhen between 2007 and 2012 were retrospectively scanned in a spatio-temporal manner by incorporating the dimensions of both time and space. The analysis detected five spatio-temporal cluster areas after the covariate of age was controlled, suggesting that breast cancer incidence was not randomly distributed in space and time. The Class 1 spatio-temporal cluster was located in southwestern Shenzhen in 2010 and included Nantou, Shahe, Shekou, and Nanshan Streets in Nanshan District, with an incidence of 54.1/100,000 (*RR* = 2.41, *LLR* = 52.84, *P* < 0.01). Four Class 2 spatio-temporal clusters were found, the most significant one of which was located in south-central Shenzhen in 2011 involving Haishan, Shatoujiao, and Yantian Streets in Yantian District with an incidence of 70.9/100,000 (*RR* = 3.25, *LLR* = 37.70, *P* < 0.01). The other three Class 2 spatio-temporal clusters were as follows: Luohu District in 2012, Bantian and other new developing streets in 2011, and Futian District in 2011. The spatio-temporal cluster areas mentioned above involved individual 1-year periods and the districts covering 3–8 streets. The distribution clusters regarding the parameters of space and time are shown in Table 2 and Figure 4.

**Table 2:** **Spatio-temporal clusters of female breast cancer incidence in Shenzhen, 2007-2012**

**Cluster**	**Year**	**Location (District)**	***RR***	***LLR***	***P*** **value**	**WASR**
						**(per 100,000)**
Class 1	2010	Yuehai, Nantou, Shahe, Shekou, Nanshan	2.41	52.84	0.001	54.1
Class 2	2011	Haishan, Shatoujiao, Yantian	3.25	37.70	0.001	70.9
		Bantian, Buji, Longhua	1.74	22.30	0.001	41.5
		Shatou, Futian, Lianhua, Xiangmihu, Huafu	1.70	21.19	0.001	40.7
	2012	Dongmen, Nanhu, Guiyuan, Sungang, Cuizhu, Huangbei, Yuanling, Nanyuan	1.85	30.99	0.001	44.1

RR, relative risk; LLR, log likelihood ratio; WASR, the age-standardized rate according to the world standard population.

## Discussion

Using geographic information system (GIS)-based spatial statistics, this analysis identified areas with high incidences of female breast cancer that should be emphasized in future cancer prevention and control. The increasing temporal trend also suggested that more attention should be paid to this disease in Shenzhen.

The identification of spatial clusters of cancer occurrence has been regarded as a useful instrument in detecting locations with a high risk of the disease. A few previous studies have compared two widely used cluster spatial methods, spatial scan statistics and local Moran’s *I*, in identifying spatial clusters of a specific disease and suggested that these two spatial analytical techniques were complementary to each other and should be used jointly rather than separately [13,14]. Using this strategy, our analysis indicated a nonrandom distribution of female breast cancer incidence in Shenzhen and revealed the presence of high-risk areas for breast cancer in eastern Luohu District and Yantian District. However, at present, few epidemiologic studies have been conducted to investigate the underlying reasons for the spatial distribution of breast cancer in Shenzhen, and future studies are warranted.

The identification of a high-risk area for the incidence of a disease is usually followed by studies aimed at examining the underlying causal mechanisms [15]. Although much remains unknown regarding the underlying reasons for the observed spatial clusters in Shenzhen, there is sufficient evidence from previous studies [6,7] to argue that the breast cancer cases were most likely influenced by a complex combination of risk factors, including certain genetic, environmental, and socio-economic factors; more importantly, the interaction of these factors might have played an important role.

It should be noted that although the incidence of breast cancer in Shenzhen was lower than the national and global averages, an increasing trend was observed in recent years [16]. A variety of factors can potentially affect the temporal trend in the breast cancer incidence in Shenzhen. In addition to genetic factors, changes in environmental and dietary factors, such as increasing air pollution, greater consumption of a more popular western diet, and less physical exercise, might help to explain the observed increasing trend [17,18]. Another possibility is the improvement in medical services and the quality of the data. Along with socio-economic development, more women underwent annual breast mammographic screening, increasing the possibility of detecting cancer; the cancer registry system also provided better cancer incidence data in more recent years [14].

In 2008, the project of early detection and treatment of cancers was initiated in Shenzhen, focusing on breast and cervical cancers. Gradually, a breast cancer-screening network was established to cover the entire city to build a highly compliant community-based screening program for breast cancer [19]. Along with the promotion of breast cancer screening in the community, more patients with potential breast cancer and precancerous lesions who were not found in previous opportunistic screenings by medical institutions have been successfully diagnosed and treated in a timely manner. In recent years, the project has led to a gradual reduction in the number of newly diagnosed patients with breast cancer, as well as a small decrease in the number of patients with breast cancer that has spread to surrounding areas. This situation partly explains the reason why short-term fluctuations existed in the spatio-temporal clusters of breast cancer incidence in Shenzhen.

The development of spatio-temporal epidemiology provided a good tool for the quantitative analysis of data on the environment and disease surveillance [20,21]. The results from this study have some important public health implications. The spatial and temporal distribution of the incidence of breast cancer provides not only important epidemiologic clues to cancer etiology for primary prevention but also a scientific basis for the appropriate allocation of health resources.

A few limitations should be noted. Our spatial and temporal analyses were based on the breast cancer incidence data. The incidence data could be influenced by access to medical care, diagnostic techniques, the quality of the cancer registration data, and so on. Differences in the cancer incidence among different areas can result from differences in the geographic distribution of these health care access and data quality variables as well as from differences in etiologic factors of the disease. In addition, there is a considerable latency time lag between the exposures to these risk factors and the occurrence of the cancer; however, none of these factors were considered in our analysis. Therefore, the results of this study should be interpreted with caution.

## Conclusion

In summary, this study identified a statistically significant cluster of breast cancer incidence in Shenzhen, including eastern Luohu District and Yantian District, and an increasing temporal trend was observed in recent years. Additional studies are required to examine the underlying reasons for this spatio-temporal pattern of breast cancer incidence in Shenzhen.
